# Exploring the Potential of a New Nickel(II):Phenanthroline Complex with L-isoleucine as an Antitumor Agent: Design, Crystal Structure, Spectroscopic Characterization, and Theoretical Insights

**DOI:** 10.3390/molecules30132873

**Published:** 2025-07-06

**Authors:** Jayson C. dos Santos, João G. de Oliveira Neto, Ana B. N. Moreira, Luzeli M. da Silva, Alejandro P. Ayala, Mateus R. Lage, Rossano Lang, Francisco F. de Sousa, Fernando Mendes, Adenilson O. dos Santos

**Affiliations:** 1 Center for Sciences of Imperatriz, Federal University of Maranhão—UFMA, Imperatriz 65900-410, MA, Brazil; jayson.cabral@discente.ufma.br (J.C.d.S.); joao.gon@ufma.br (J.G.d.O.N.); nunes.ana@discente.ufma.br (A.B.N.M.); luzeli.moreira@ufma.br (L.M.d.S.); mateus.lage@ufma.br (M.R.L.); 2 Department of Physics, Federal University of Ceará—UFC, Fortaleza 65455-900, CE, Brazil; ayala@fisica.ufc.br; 3 Institute of Science and Technology, Federal University of São Paulo—UNIFESP, São José dos Campos 12231-280, SP, Brazil; rossano.lang@unifesp.br; 4 Institute of Exact and Natural Sciences, Federal University of Para—UFPA, Belém 66075-110, PA, Brazil; ffs@ufpa.br; 5 Department Biomedical Laboratory Sciences, ESTEC-Coimbra Health School, Polytechnic Institute of Coimbra, 3046-854 Coimbra, Portugal; fjmendes@estesc.ipc.pt

**Keywords:** nickel(II) complex, crystal growth, DFT calculations, Hirshfeld surfaces, vibrational spectroscopy, cytotoxicity

## Abstract

This study presents the synthesis, physicochemical characterization, and biological evaluation of a novel ternary nickel(II) complex with isoleucine and 1,10-phenanthroline ligands, [Ni(Phen)(Ile)_2_]∙6H_2_O, designed as a potential antitumor agent. Single-crystal X-ray diffraction revealed a monoclinic structure (C2-space group) with an octahedral Ni(II) coordination involving Phen and Ile ligands. A Hirshfeld surface analysis highlighted intermolecular interactions stabilizing the crystal lattice, with hydrogen bonds (H···H and O···H/H···O) dominating (99.1% of contacts). Density functional theory (DFT) calculations, including solvation effects (in water and methanol), demonstrated strong agreement with the experimental geometric parameters and revealed higher affinity to the water solvent. The electronic properties of the complex, such as HOMO−LUMO gaps (3.20–4.26 eV) and electrophilicity (4.54–5.88 eV), indicated a charge-transfer potential suitable for biological applications through interactions with biomolecules. Raman and infrared spectroscopic studies showed vibrational modes associated with Ni–N/O bonds and ligand-specific deformations, with solvation-induced shifts observed. A study using ultraviolet–visible–near-infrared absorption spectroscopy demonstrated that the complex remains stable in solution. In vitro cytotoxicity assays against MCF-7 (breast adenocarcinoma) and HCT-116 (colorectal carcinoma) cells showed dose-dependent activity, achieving 47.6% and 65.3% viability reduction at 100 μM (48 h), respectively, with lower toxicity to non-tumor lung fibroblasts (GM07492A, 39.8%). Supporting the experimental data, we performed computational modeling to examine the pharmacokinetic profile, with particular focus on the absorption, distribution, metabolism, and excretion properties and drug-likeness potential.

## 1. Introduction

Nickel complexes are coordination compounds, which are versatile due to their geometry and preference for forming six bonds. Ni(II) is commonly found in aqueous solutions and within the human biological system. This makes it highly favorable for interactions with several organic ligands [1]. Additionally, Ni(II) complexes have applications in areas such as nonlinear optics, owing to their high stability at elevated temperatures and ability to emit light in the visible region, modulate optical signals, and generate harmonics. They are also used in the manufacture of thermochemical devices due to the abundance of active sites on this metal ion [2,3].

The applications of Ni(II) complexes expand significantly when coordinated to bidentate ligands such as 1,10-phenanthroline (Phen), given the well-known ability of this chelating agent to interact with transition metals. Its heterocyclic structure and nitrogen atoms facilitate electron acceptance, leading to the formation of metal–organic compounds [4], while strong deoxyribonucleic acid (DNA) binding and cleavage result in notable biological activity [5]. De Souza Junior et al. and De Oliveira Neto et al. highlight, respectively, the bactericidal effects against *Staphylococcus aureus*, *Enterococcus faecalis*, and *Pseudomonas aeruginosa* and the antineoplastic activity against human mammary adenocarcinoma and cervical carcinoma tumor cells using Phen complexes involving transition metals [6,7]. Furthermore, Nath et al. emphasize the apoptosis-inducing potential of a nickel(II):Phen complex in lymphatic cancer cells [8].

Amino acids can be added to transition metal complexes with Phen to enhance or adjust the biological properties of these compounds. Souza Junior et al., De Oliveira Neto et al., Ramos et al., and Pereira et al. reported promising cytotoxic results of copper(II):Phen complexes with L-glutamine, L-glycine, L-serine, and L-tyrosine against prostate cancer cells (PC-3), glioblastoma (SNB-19), and colorectal carcinoma (HCT-116) [9,10,11,12]. Additionally, Wei et al. confirmed the DNA-binding capacity of Ni(II) complexes with Phen and glutamine [13].

Another important amino acid widely used in complexation is L-isoleucine (Ile). Its side chain and chemical structure, featuring an amino group (–NH_2_) and a carboxylic group (–COOH), make it a promising chelating agent for coordination with metal ions [14]. For humans, it is an essential amino acid involved in protein and fatty acid metabolism, as well as aiding in wound healing through muscle fiber recovery. The deficiency of this macromolecule can lead to hypoglycemia [15]. Furthermore, its antioxidant potential is reported to reduce enzymatic browning in foods and oxidative stress in the human body, preventing diseases such as Alzheimer’s, diabetes, and atherosclerosis [16]. Rao et al. investigated the ability of a copper(II) complex containing Ile to effectively cleave DNA, while Farhangian and Kharat reported that this amino acid complexed with platin(II) inhibits ovarian cancer cells more effectively than cisplatin [17,18].

Motivated by this context, this paper reports a new ternary [Ni(Phen)(Ile)_2_]∙6H_2_O complex, synthesized via the slow solvent evaporation method, with its structure solved using single-crystal X-ray diffraction (XRD). Furthermore, density functional theory (DFT) calculations, considering solvation effects, were performed to analyze the inter- and intramolecular modes detected in experimental Fourier transform infrared (FT-IR) and Raman spectra. The stability of the complex in solution was investigated using ultraviolet–visible–near-infrared (UV–Vis–NIR) spectroscopic measurements. Additionally, a comprehensive qualitative and quantitative study of the interactions between the compound’s chemical species was carried out using Hirshfeld surfaces and their analogues. Additionally, a cell viability assay was conducted using two MCF-7 (breast cancer) and HCT-116 (colorectal cancer) tumor cell lines and the GM07429A (lung fibroblast) healthy cell line to assess the complex’s cytotoxic effects. Finally, the biological experiments were complemented with in silico pharmacokinetic data obtained from the analysis of adsorption, distribution, metabolism, and excretion (ADME) parameters.

## 2. Results and Discussion

### 2.1. Crystallization and Determined Structure by Single-Crystal XRD

**Figure 1:**
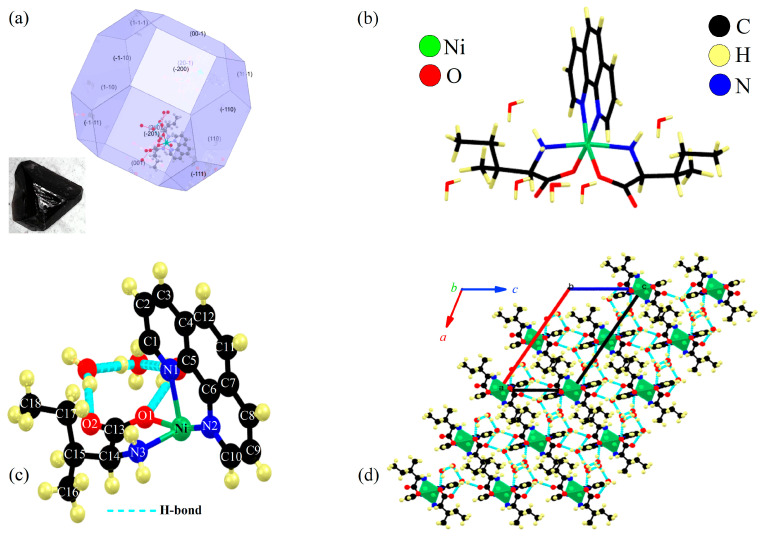
(**a**) Morphology and growth habit of [Ni(Phen)(Ile)_2_]∙6H_2_O crystal. (**b**) Molecular structure. (**c**) Symmetric monomer with labels and hydrogen bonds (dashed light blue line). (**d**) Super unit cell (3 × 3 × 3) and its primitive cell along the *b*-axis.

The complex was successfully crystallized at basic pH via the slow evaporation method, as previously described. As shown in Figure 1a, the sample exhibited a polygonal morphology with well-defined facets and dark purple coloration. Furthermore, the calculated crystal habit corroborates the experimental data, revealing a polygonal nature with 14 crystallographic planes: (-1-11), (1-10), (-1-10), (1-1-1), (001), (-201), (200), (-200), (20-1), (00-1), (11-1), (-110), (110), and (-111). However, during the solid-phase nucleation process, some planes are more favorable than others due to the lack of control over the solvent evaporation rate, which leads to surface defects. Additionally, the modeled morphology displays a well-defined polygonal crystal habit with predominant (001) and (110) crystal faces. This geometry is directly associated with the symmetric molecular arrangement in the crystal lattice. A quantitative analysis of the modeled habit revealed that (001) and (110) faces comprise 72% of the total surface area, exhibiting interfacial angles of 84° ± 3°. This structural arrangement directly correlates with the observed hydrogen-bonding lattice (O1···H–O* = 2.035 Å, 160.14°) and π∙∙∙π interactions (3.8 Å). This anisotropic growth not only contributes to the stability of the crystal but also leads to an increase in the hydrophilicity of the complex. For structure determination, a single crystal with average dimensions around 0.697 × 0.512 × 0.318 mm^3^ was used.

Figure 1d illustrates a representative diagram of the supramolecular assembly, derived from a 3 × 3 × 3 supercell. The stability of assembly primarily arises from hydrogen bonds involving free H_2_O molecules and the carboxyl group of the Ile ligand. Additionally, weak π∙∙∙π stacking between the aromatic rings of Phen molecules (with a centroid-to-centroid distance of ~3.8 Å) contributes to the overall stability. Together, these interactions minimize steric clashes, promoting long-range order and the observed polyhedral crystalline habit. Furthermore, the differing physicochemical properties of the ligands lead to a slightly distorted bipyramidal geometry around the coordination sphere.

Based on the data obtained by single-crystal XRD, it could be noticed that the complex has an empirical formula of C_24_H_44_N_4_NiO_10_ and a molecular mass of 607.24 g/mol. At room conditions (300 K), it crystallizes with monoclinic symmetry, C2($C_{2}^{3}$)-space group, and has two formulas of [Ni(bis-isoleucine)(1,10-phenanthroline)](H_2_O)_6_ per unit cell (*Z* = 2). The lattice parameters obtained are as follows: *a* = 18.0810(1) Å, *b* = 10.1111(1) Å, *c* = 10.0475(1) Å, *α* = 90°, *β* = 122.137(2)°, *γ* = 90°, and *V* = 1555.4(3) Å^3^. The Flack parameter of 0.16(3) confirms the correct configuration assignment for L-Ile within the crystal structure. The slight deviation from zero is likely to arise from either minor crystal imperfections or systematic measurement errors, possibly influenced by uncontrolled solvent evaporation during crystallization. This value falls within the acceptable range (<0.2) for enantiopure compounds, aligning with findings from similar metal–amino acid complexes reported in the literature [19,20,21]. A summary of the structure and refinement parameters can be found in Table 1.

To ensure the reproducibility, reliability, and crystalline phase purity of the synthesized crystal, additional syntheses were conducted, and the structure was investigated by powder XRD. Employing the previously solved structure deposited in the Cambridge Structural Database (CSD entry 2453842), the experimental diffractogram presented in Figure S1 was refined using the Rietveld method. The refined pattern shows that [Ni(Phen)(Ile)_2_]·6H_2_O crystallizes with identical crystallographic parameters (symmetry, space group, and *Z*) as those shown in Table 1. Also, one observes a good concordance of the following unit cell parameters: *a* = 18.072(8) Å, *b* = 10.109(7) Å, *c* = 10.048(4) Å, *β* = 122.11(2)°, and V = 1554.99(4) Å^3^. This concordance confirms the crystalline phase purity, reproducibility, and reliability of the synthesis.

Figure 1b,c show the chemical structure and the symmetrical unit of the [Ni(Phen)(Ile)_2_]∙6H_2_O crystal with the labels. The coordination sphere around the nickel atom is coordinated by two oxygen atoms and two nitrogen atoms from the carboxylate and amine groups of the two Ile molecules and two nitrogen atoms of the Phen molecule, forming a distorted square bipyramidal geometry. In the structure, one of the Ile molecules has an oxygen atom of the carboxylate group (Ni–O1) in the axial position above the equatorial plane, while the amine nitrogen (Ni–N3) is below this plane. The other Ile molecule exhibits opposite positions, creating an asymmetric plane that forms *cis* isomerism. Furthermore, as related in similar studies [22,23], the Ni(II) shows a preference for forming octahedral structures, which is why it also binds to the nitrogen atoms of the Phen ligand. Additionally, each monomer has six free H_2_O molecules that stabilize the crystal lattice through intermolecular contacts, as shown in Figure 1d.

Table A1 and Table A2 (Appendix A section) display the bond lengths and angles for the complex. The bond lengths between the nickel atom and the amino acid atoms are 2.082(1) Å (Ni–N3/Ni–N3′) and 2.054(2) Å (Ni–O1/Ni–O1′), while the bond lengths to the nitrogen atoms of the Phen ligand are 2.080 Å (Ni–N1) and 2.131(6) Å (Ni–N2). These values are consistent with those reported for other nickel complexes featuring similar N, O-coordination environments, such as bis(*β*-alaninato)nickel(II) [21] and bis(L-histidinato)nickel(II) monohydrate [23]. The bond angles are 80.36(8)° (O1–Ni–N3/O1′–Ni–N3′) for the two Ile molecules and 78.30(4)° for the Phen ligand. The similarity in the bond lengths and angles for both ligands is characteristic of octahedral geometries. Furthermore, Table A3 (Appendix A section) lists the hydrogen bonds in the crystal structure of [Ni(Phen)(Ile)_2_]∙6H_2_O. The data highlight the key role of the Ile ligand as the primary provider of hydrogen-bonding donor/acceptor sites, facilitating specific interactions with the free H_2_O molecules.

### 2.2. Analysis of Intermolecular Interactions by Hirshfeld Surfaces and Their Analogs

**Figure 2:**
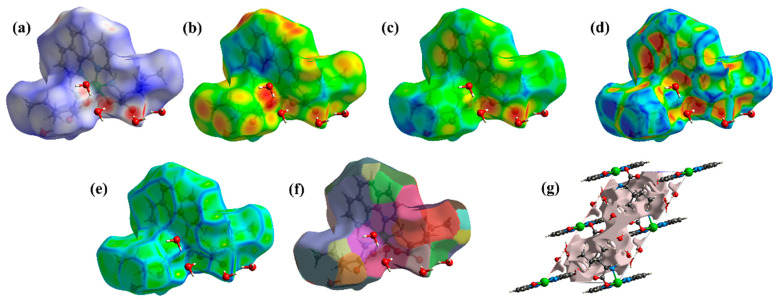
Hirshfeld surface mapping in terms of (**a**) *d_norm_,* (**b**) *d*_e_, (**c**) *d_i_,* (**d**) shape index, (**e**) curvedness, (**f**) fragment patch, and (**g**) crystal voids. Warm color patterns represent short, intense interactions, while cool color patterns indicate distant, weaker ones.

To complement the structural data, a comprehensive study of the intermolecular interactions in the [Ni(Phen)(Ile)_2_]∙6H_2_O crystal lattice was performed using Hirshfeld surfaces [24,25]. Figure 2a shows the three-dimensional map in terms of the *d*_norm_ function, where the tricolor pattern represents the intensity of each contact: red, contacts with distances smaller than *r*_vdW_; white, contacts with distances similar to *r*_vdW_; and blue, contacts with distances larger than *r*_vdW_ [26]. The reddish regions are located around the O and H atoms, indicating sites where strong intermolecular interactions, such as hydrogen bonds, predominate. Additionally, slightly weaker contacts are represented by the whitish areas centered around the aromatic ring of Phen and the N and C atoms of the Ile ligands. These interactions originate from *π*−*π* bonds due to the resonance effect in the cyclic regions involving the pyridine rings and the contact established with neighboring organic moieties. Furthermore, the Hirshfeld surfaces plotted as a function of *d*_i_ (Figure 2b) and *d*_e_ (Figure 2c) complement these results. The reddish and yellowish areas in Figure 2b, observed mainly over the O and H atoms of the [Ni(Phen)(Ile)_2_]∙6H_2_O crystal, represent donor sites of intermolecular interactions. At the same time, in Figure 2c, these regions, highlighted in warm tones over the H_2_O molecules, functional groups of the structure, and pyridine rings, characterize sites that act as contact acceptors [27].

Figure 2d,f show the complex’s shape index, curvedness, and fragment path. These surfaces are qualitative computational analyses that provide structural data corresponding to the molecules’ topology. The shape index shown in Figure 2d reveals the regions (orange and yellow) from the most conducive to the strongest intermolecular contacts, in which the molecules are stacked together to form the crystal lattice. Similarly, the curvedness (Figure 2e) delimits the interaction sites, with the flat greenish regions representing the molecular stacking centers. Furthermore, the blue contour indicates where the tendency for hydrogen bonding with neighboring monomers occurs. Moreover, the regions mapped by different colors in Figure 2f are associated with equivalent interaction sites involving two [Ni(Phen)(Ile)_2_]∙6H_2_O units. In other words, they correspond to molecular docking zones that provide structural ordering and stability to the crystal lattice [28].

Additionally, a theoretical experiment was conducted using electron density isosurfaces to quantify the void volume of the complex unit cell, as illustrated in Figure 2g. The calculated data indicate a void percentage of 11.7% (181.52 Å^3^) and a surface area of 626.87 Å^2^. These values suggest that the free area is classified as regular and does not compromise the lattice energy between the chemical species of the crystal system. Moreover, these voids can be occupied by introducing impurities or dopants to adjust or improve the specific physicochemical properties of the material.

**Figure 3:**
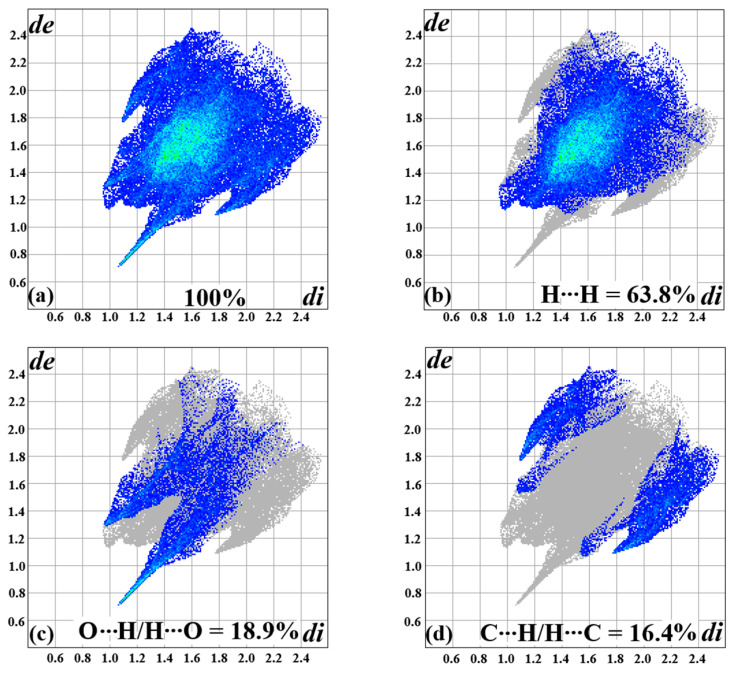
Two-dimensional fingerprint graphs of intermolecular interactions: (**a**) total, (**b**) H···H, (**c**) O···H/H···O, and (**d**) C···H/H···C.

To provide more details about the intermolecular interactions of the complex, a quantitative analysis was performed from 2D fingerprint plots, as shown in Figure 3a–d. The graphs are generated in the form of stratified histograms (Figure 3b–d) from a cumulative pattern involving all interactions (Figure 3a) represented by blue dots distributed from the properties of *d*_e_ and *d*_i_. It is important to highlight that the points observed in areas of low values suggest a strong nature of the quantified intermolecular interactions [29]. The calculated data indicates that the H····H, O···H/H···O, and C···H/H···C interactions account for approximately 99.1% of the contacts formed between the monomers. The remaining percentage corresponds to weaker interactions, such as O···C/C···O and O···O. In summary, hydrogen bonds are the main contacts providing structural stability in the complex. However, intermediate contacts also contribute to the order of the molecular units.

### 2.3. Computational Studies

**Figure 4:**
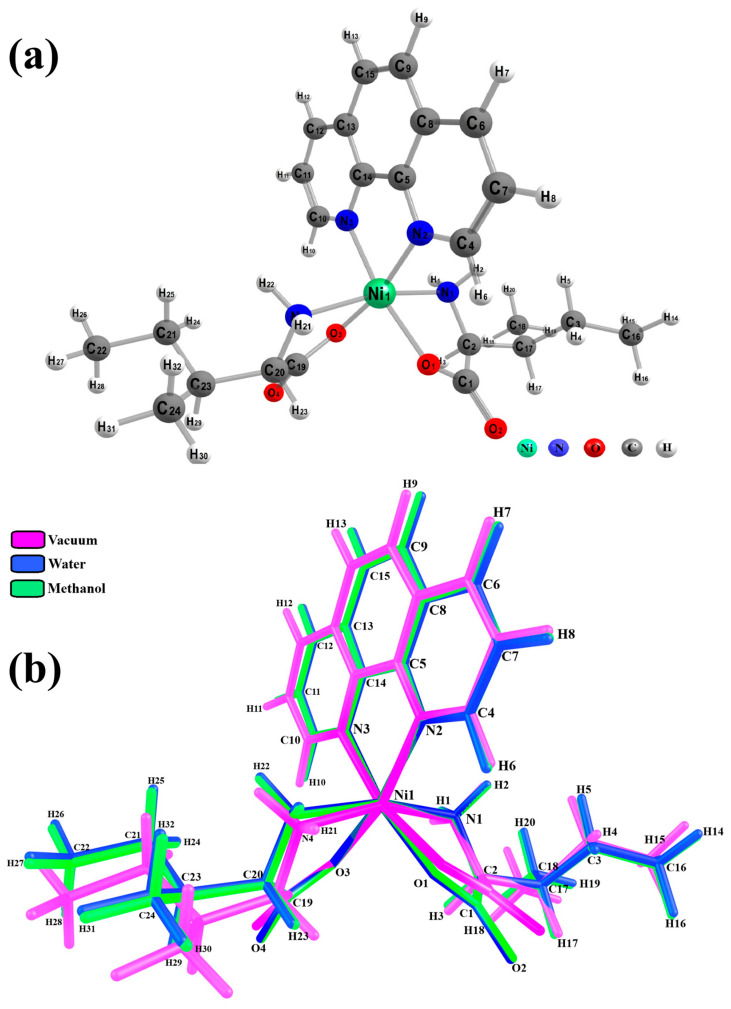
(**a**) Optimized geometry of the [Ni(Phen)(Ile)_2_] complex obtained at the PBE1PBE/6–311++G(d,p)/SDD level of theory, with labels, and (**b**) superposition of the [Ni(Phen)(Ile)_2_] chemical structure under vacuum (purple), water (blue), and methanol (green) conditions.

From the computational calculations using DFT with the PBE1PBE functional and the 6–311++G(d,p)/SDD basis set, we obtained the optimized geometry of the complex under a solvation effect (Figure 4a) and in the gas phase (Figure 4b), disregarding the free H_2_O molecules present in the complex. A few visible structural differences were observed in the [Ni(Phen)(Ile)_2_] monomer under the conditions analyzed. However, a thorough comparative study of the geometric parameters, including bond lengths and angles, revealed slight differences, as shown in Table A4.

The RMSD results reveal that the main differences in bond lengths occur in the Ni01–O1′ (0.0296 Å) and Ni01–O1 (0.0296 Å) metal–Ile bonds, as well as in the Ni01–N1 (0.0480 Å) metal–Phen bond under vacuum conditions, though most differences in this environment remain below 0.1 Å, indicating good agreement between the theoretical and experimental results. Under the solvation effect, the data shows even closer alignment with the experimental values, particularly in cases of perfect matching (0.0000 Å) for Ni01–N2 and O2–C13 in water and O2–C13 in methanol, with the largest deviation observed being just 0.0360 Å for both solvents, further validating the accuracy of calculations associated with the solvents.

Similar trends are noticed in the bond angle comparison between the theoretical and experimental parameters. Under vacuum conditions, the O1′–Ni01–N1 (8.1458°) and O1′–Ni01–N2 (7.9195°) bonds showed the largest RMSD deviations. In solvated environments, the most significant differences occurred for the O1′–Ni01–N1 (5.0063°) and O1–Ni01–N1 (4.4547°) bonds in water, while in methanol, the O1′–Ni01–N2 (4.5467°) and O1–Ni01–N2 (4.6952°) angles exhibited the greatest variations. As illustrated in Figure 4b, the best agreement between the computational results and experimental structural data was achieved under solvation conditions, with minimum differences between the water and methanol solvents, confirming the accuracy of the calculations in these media.

The Gibbs free energy values calculated at 298 K indicate good affinity of the complex to the two solvents used but with higher affinity to water, confirming strong solute–solvent affinity mediated by electrostatic interactions. The calculations confirm that both water and methanol are suitable solvents for the dissolution of the [Ni(Phen)(Ile)_2_] complex, but the mixture of solvents ensures improved dissolution of the reactants.

**Figure 5:**
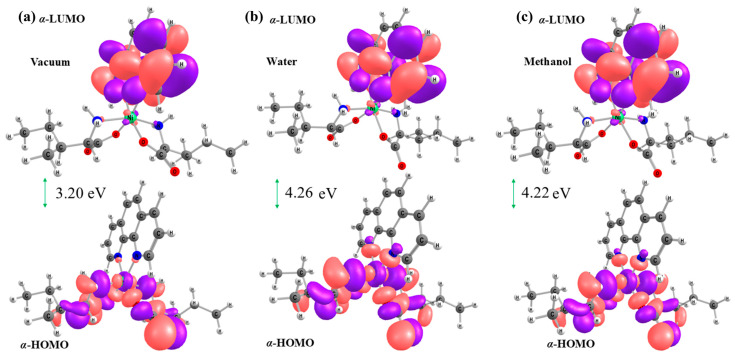
HOMO and LUMO orbital plots calculated using the PBE1PBE/6–311++G(d,p)/SDD level of theory in (**a**) vacuum, (**b**) water, and (**c**) methanol for the [Ni(Phen)(Ile)_2_] complex. Pink surface—negative region and Purple surface—positive region.

DFT calculations employing PBE1PBE enabled the characterization of the electronic properties of the [Ni(Phen)(Ile)_2_] complex through reactivity indices (Table 2), including frontier molecular orbitals (*α*-HOMO and *α*-LUMO) and the energy gap (Figure 5) [30,31,32]. Under vacuum conditions, the *α*-HOMO (−5.94 eV) is primarily localized on the metal center and Ile rings, while the *α*-LUMO (−2.74 eV) is predominantly distributed over the Phen ligand. Solvation effects induce significant energy shifts: in water, the values are −6.53 eV (*α*-HOMO) and −2.27 eV (*α*-LUMO), while in methanol, they are −6.51 eV (*α*-HOMO) and −2.28 eV (*α*-LUMO), maintaining the same electron distribution pattern. These results demonstrate that the complex exhibits intrinsic electronic stability and potential for efficient interactions with biological systems or other molecular targets, particularly through the Phen ligand [33].

The frontier orbital analysis yielded HOMO−LUMO gap values of 3.20 eV (vacuum), 4.26 eV (water), and 4.22 eV (methanol), classifying the solvated complex as a dielectric system (gap > 4 eV) with adjusted electronic transition capabilities [33]. These findings align with dipole moment data, which increased from 18.16 D (vacuum) to 23.88 D (water) and 23.68 D (methanol), demonstrating increased charge redistribution in the solvents. This behavior indicates that intramolecular charge transfer (between positive/negative centers) is amplified in solvated environments, being able to promote the [Ni(Phen)(Ile)_2_] complex interaction with biomolecular targets. Furthermore, solvent-induced molecular stabilization is confirmed by the >1.0 eV gap enlargement compared to vacuum, reflecting greater electronic stability in solvated states.

The calculated chemical reactivity indices, which include the ionization potential (*IP* < 6.0 eV), electron affinity (*EA* > 2.0 eV), hardness (*η*), softness (*ς*), chemical potential (*μ*), electronegativity (*χ*), and electrophilicity (*ϖ*), demonstrated that the [Ni(Phen)(Ile)_2_] complex exhibits suitable electronic properties for pharmacological applications, featuring low ionization energy and high electron-accepting capability, indicating an optimal balance between molecular stability and controlled reactivity, crucial for effective interactions with biomolecules. The comprehensive analysis of these parameters reveals that the system possesses well-balanced electronic properties, with *IP* and *EA* values within the optimal ranges for bioactive compounds, along with significant charge transfer capacity, supporting its potential as a promising pharmaceutical agent candidate.

Additionally, chemical hardness (*η*) characterizes the ligands’ basicity and the metal ion’s acidity, while softness (*ς*) describes the ease of the charge transfer [34]. These parameters govern the system’s polarizability. The maximum hardness was observed in water (*η* = 2.13), whereas the softness remained constant (*ς* = 1.06) in both water and methanol, indicating that the molecule is susceptible to polarization, especially in aqueous environments.

The high electronegativity (χ = 4.40 eV) and corresponding negative chemical potential (μ = −4.40 eV), found in both solvents, reinforce the ability of the complex to interact with biomolecules. This promotes interactions with other molecules, facilitating spontaneous reactions [29]. Another key descriptor of biological activity is electrophilicity (*ϖ*), as the electron acceptance tendency favors interactions with biomolecular targets [12]. The obtained values, 5.88 eV (vacuum), 4.54 eV (water), and 4.58 eV (methanol), suggest that the complex exhibits potential biological activity. When compared to previously reported coordination compounds with similar antitumor activity [35,36], this value exhibited a higher index, further supporting its enhanced biological efficacy against pathogens.

**Figure 6:**
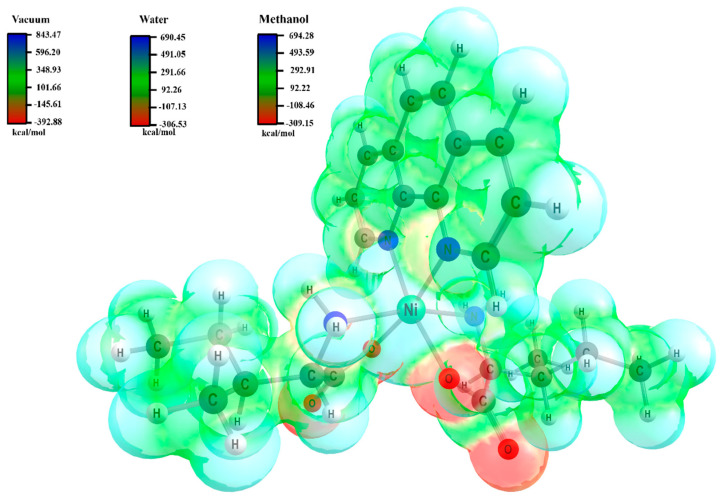
ESP map of the [Ni(Phen)(Ile)_2_] complex calculated using the PBE1PBE/6–311++G(d,p)/SDD level of theory in vacuum, water, and methanol, with a potential range of −0.02 to 0.06.

DFT calculations in vacuum and solvation media (water and methanol) were performed to generate the electrostatic potential (ESP) map of the [Ni(Phen)(Ile)_2_] complex, identifying regions with the highest interaction potential and electron density (see Figure 6) [37]. The red-colored regions, predominantly localized on the oxygen atoms of Ile, represent nucleophilic (electron-rich) sites with negative potentials, indicating a tendency to donate electrons and interact with positive regions of other molecules. This is supported by the following calculated values: −392.88 kcal/mol (vacuum), −306.53 kcal/mol (water), and −309.15 kcal/mol (methanol). Conversely, blue/green surfaces correspond to intermediate-to-positive electrostatic potentials, distributed around the metal ion and Phen rings, as evidenced by the maximum energies of 843.47 kcal/mol (vacuum), 690.45 kcal/mol (water), and 694.28 kcal/mol (methanol). Overall, no significant variations were observed in the ESP surfaces across the different environments.

### 2.4. Spectroscopic Analysis Combined with DFT Calculations

**Figure 7:**
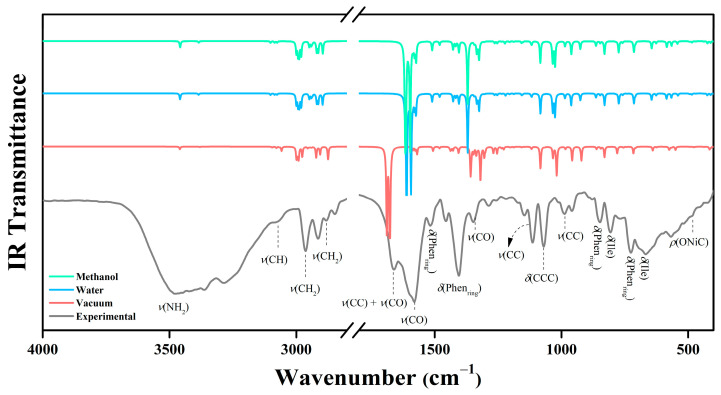
Experimental IR spectrum of [Ni(Phen)(Ile)_2_]∙6H_2_O powdered crystal compared with the calculated spectra in vacuum, water, and methanol.

**Figure 8:**
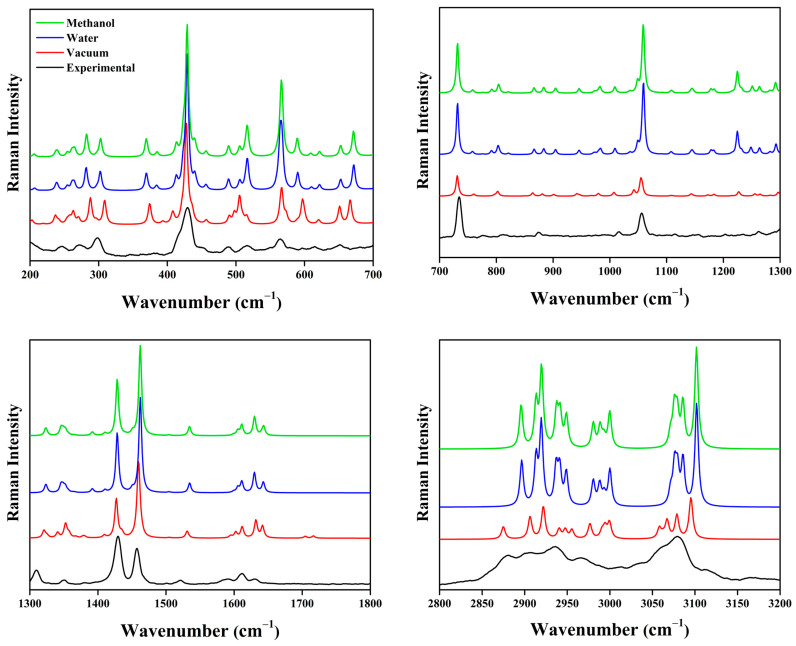
Experimental Raman spectrum of [Ni(Phen)(Ile)_2_]∙6H_2_O powdered crystal compared with the calculated spectra in vacuum, water, and methanol.

Vibrational spectroscopy (FT-IR/Raman) was employed to validate the XRD-derived structure of [Ni(Phen)(Ile)_2_] and characterize its functional groups. The experimental spectra were compared with the PBE1PBE/6-311++G(d,p)/SDD calculations (scaling factors: 0.9594–0.9812) [38] in vacuum, water, and methanol environments (Table A5, Figure 7 and Figure 8). The vibrational assignments used the VMARD ≥ 9% criteria [39].

The symmetric stretching vibrations at 3479 and 3361 cm^−1^ correspond to the broad band in the experimental IR spectrum. These vibrations are observed in the theoretical spectra in vacuum at 3459 and 3380 cm^−1^ and in the solvation calculations with water (3458 and 3385 cm^−1^) and methanol (3457 and 3384 cm^−1^). These modes are associated with the N–H bonds of the Ile molecule. In the IR spectrum and in the Raman spectrum (Figure 8), the bands at 3058 cm^−1^ and at 3067 cm^−1^, respectively, are attributed to C–H group vibrations of the Ile and Phen ligands. These modes also appear at 3075 cm^−1^ in both water and methanol. Such regions show minimal contributions from the solvation theoretical calculations to the structural bonding patterns (Table A4).

The vibrational IR modes at 1660 cm and 1581 cm^−1^ and the Raman modes at 1630 and 1610 cm^−1^ are associated with the C=O double bond stretching of the amino acid. These vibrations correlate with the theoretical calculations of 1690 and 1679 cm^−1^ (vacuum), 1614 and 1592 cm^−1^ (water), and 1615 and 1594 cm^−1^ (methanol). From 1517 cm^−1^ (IR) and 1521 cm^−1^ (Raman), the ring deformation modes of Phen become evident, also appearing under solvation effects at 1510 cm^−1^ (both in water and methanol). In general, the vibrational modes associated with the Phen ligand exhibit characteristic shifts that vary according to the metal center’s coordination sphere and the complex geometry. These variations are attributed to the physicochemical properties of the transition metals, such as ionic radius, electron affinity, and electronegativity, as observed in analogous complexes described in the literature [7,40,41]. Additionally, as the wavenumbers decrease, the discrepancies between the experimental and theoretical data tend to increase. This behavior suggests that the solvation medium used in the simulations affects the intermolecular interactions, leading to slight distortions in the complex geometry.

In the Ile molecule, vibrational modes corresponding to C–C bond scissoring and C–H stretching are observed at 1456 cm^−1^ (IR) and 1457 cm^−1^ (Raman), while C–O group stretching appears at 1348 cm^−1^ (IR) and 1349 cm^−1^ (Raman). In the spectral range between 1147 and 987 cm^−1^ (IR) and between 1147 and 998 cm^−1^ (Raman), fingerprint modes of N–C interaction scissoring, C–C stretching, and pyridine ring deformations of the Phen molecule are identified. These vibrational signatures demonstrate the structural complexity of the system, revealing how different functional groups contribute to the overall vibrational spectrum. Furthermore, the vibrational modes associated with the C–O and N–C groups exhibited significant shifts compared to the free Ile ligand [42]. These changes are attributed to the coordination of these groups to the Ni(II), resulting in alterations in the electron density distribution and bond force constants, as previously reported in the literature for coordination compounds [11,33].

The decrease in wavenumber leads to a significant reduction in the vibrational intensity, resulting in enhanced interactions with the metallic center due to the greater mass of nickel. Consequently, the vibrational modes observed in the 595–451 cm^−1^ spectral regions (IR and Raman) correspond to the wagging, torsion, and rocking motions of N–Ni and N–C interactions from the alkyl group of Ile coordinated to the metal. Additionally, the Ni–O and Ni–C bonds exhibit characteristic wagging and torsion vibrational modes arising from their coordination with the Phen molecule. In the experimental spectra, the bands at 486 (IR), 451 (Raman), and 420 (IR) cm^−1^ are assigned to molecular breathing modes of the aromatic system in Phen, demonstrating the influence of metal coordination on the overall vibrational dynamics of the system.

The vibrational bands observed in the Raman spectrum at 384, 372, and 246 cm^−1^ correspond to the torsion and stretching modes of Ni–O and Ni–N interactions in Ile coordinated to the transition metal, along with characteristic amino acid ring deformations. Below 200 cm^−1^, lattice vibrations are recorded involving collective motions of all constituent atoms in the metal complex. These low-frequency vibrations represent crucial interactions for the structural stability of the studied material, highlighting the significance of intermolecular contributions to the system’s overall properties.

### 2.5. Solution Stability Study Using UV–Vis–NIR Spectroscopy

**Figure 9:**
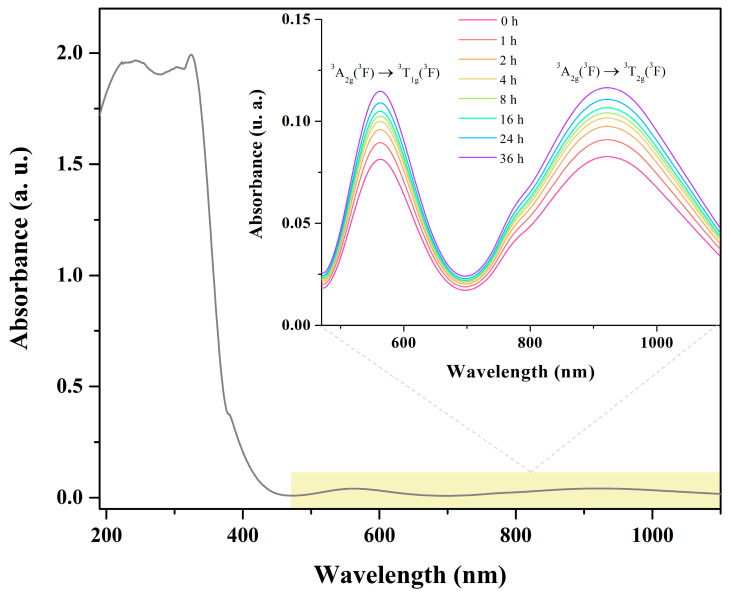
UV–Vis–NIR spectrum of the [Ni(Phen)(Ile)_2_]∙6H_2_O complex in a deionized water/ethanol (1:1) mixed solvent system, recorded in the 190–1100 nm range. Inset: time-dependent (36 h) UV–Vis–NIR spectra of [Ni(Phen)(Ile)_2_]∙6H_2_O in aqueous solution.

Figure 9 shows the molecular absorption spectrum of the [Ni(Phen)(Ile)_2_]∙6H_2_O complex in the UV–Vis–NIR region. This spectrum was obtained from powdered crystals in a 0.01 mol/L solution using a 1:1 deionized water/ethanol mixture as a solvent. The gray spectrum displays intense bands in the UV region (up to ≈ 390 nm). These are primarily attributed to *π*→π* transitions of the ligands, with the Phen ligand contributing significantly to the strong absorptions in this range [9]. Additionally, ligand-to-metal charge transfer contributions from non-bonding electron pairs of oxygen and nitrogen atoms of both Ile and Phen may also be present. In the Vis–NIR region, two characteristic bands are observed at 563 nm and 920 nm, associated with *d*−*d* electronic transitions of the Ni(II) center within an octahedral geometry (*d*^8^ configuration). Specifically, they correspond to spin-allowed transitions from the ground state ^3^A_2g_(^3^F) to the excited states ^3^T_1g_(^3^P) (≈563 nm) and ^3^T_2g_(^3^F) (≈920 nm) [41].

Additionally, time-dependent UV–Vis–NIR spectroscopic measurements were conducted during the dissolution of the complex in an aqueous medium to monitor its stability in solution (as shown in the inset of Figure 9). The recorded spectra exhibited no significant variations over the 36-h analysis period, with only a slight absorbance increase attributed to progressive material solubilization. This behavior indicates the structural stability of the [Ni(Phen)(Ile)_2_]∙6H_2_O complex in solution. Further evidence of stability was provided by the constant pH value, which remained between 9.9 and 10.0 throughout the monitoring period.

### 2.6. Antitumor Activity and Pharmacokinetic Predictions

**Figure 10:**
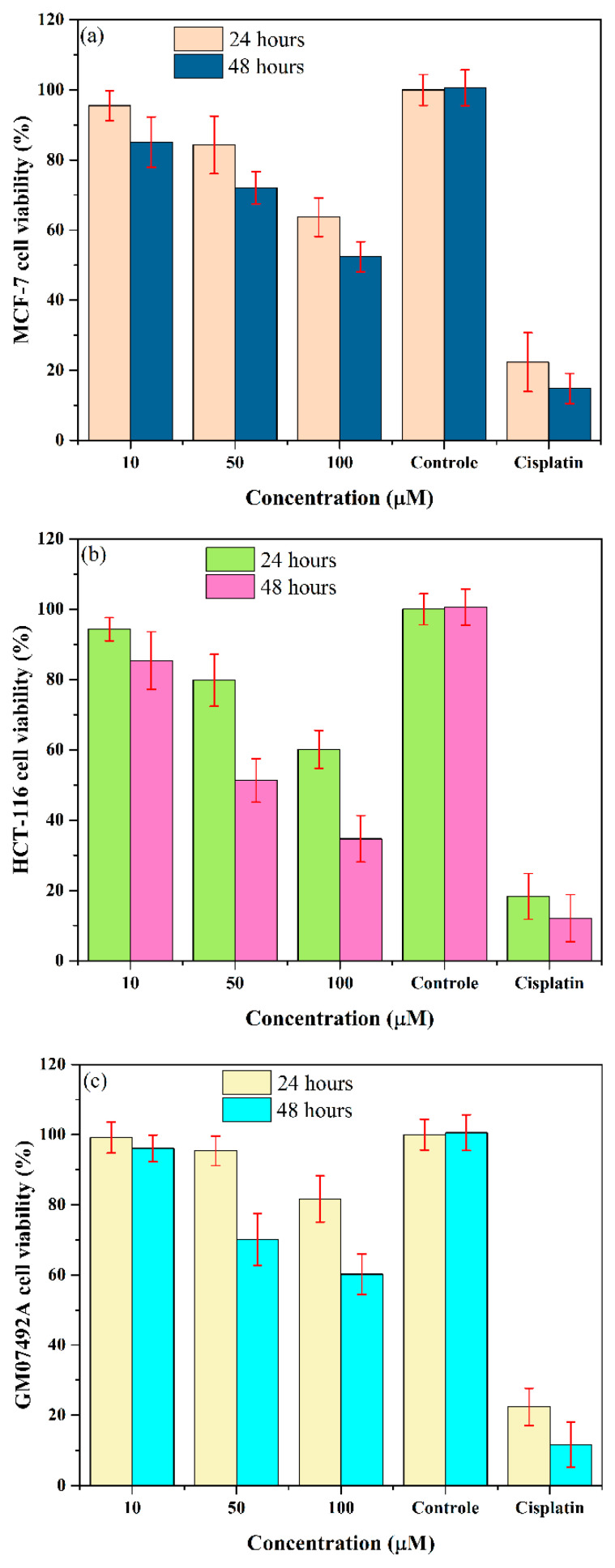
Cell viability of [Ni(Phen)(Ile)_2_]∙6H_2_O powdered crystal over 24 and 48 h period in cell lineages: (**a**) MCF-7, (**b**) HCT-116, and (**c**) GM07492A.

Building on the structural and electronic insights from previous sections, we evaluated the antitumor potential of [Ni(Phen)(Ile)_2_]·6H_2_O, focusing on its cytotoxicity, selectivity, and pharmacokinetic profile. This section explores how the unique coordination environment (octahedral Ni(II) with Phen and Ile) influences the bioactivity, leveraging experimental and computational data. Figure 10 shows the results for three cell lines: MCF-7 (breast adenocarcinoma), HCT-116 (colorectal carcinoma), and GM07492A (human lung fibroblasts) at concentrations of 10, 50, and 100 μM and with negative and positive controls (cisplatin—CP).

As shown in Figure 10a, the complex exhibited dose-dependent cytotoxicity against MCF-7 cells. A progressive inhibition of cell proliferation was observed, reaching a 47.6% viability reduction after 48 h at 100 μM concentration. Although significant, this activity was lower than cisplatin (85.2%). For the HCT-116 line (Figure 10b), the dose-response profile was similar but with notably higher efficacy (65.3% inhibition at 100 μM/48h). The higher cytotoxicity against HCT-116 (65.3% inhibition) compared to MCF-7 (47.6%) may arise from the complex’s enhanced uptake in colorectal carcinoma cells, facilitated by the hydrophobic side chain of Ile (isopropyl group), which promotes membrane penetration. In contrast, the lower activity in MCF-7 cells may result from intrinsic resistance mechanisms, including the overexpression of efflux transporters, such as P-glycoprotein and metallothioneins, as well as more robust antioxidant defenses [43,44]. These factors likely reduce the intracellular accumulation of the complex, despite its favorable DNA-binding capability via the planar aromatic system of phenanthroline.

Compared to structurally similar Ni(II)-Phen complexes in the literature (e.g., [Ni(L^1^)(Phen)]Cl_2_·2H_2_O (L^1^ = *N*^1^*E*,*N*^2^*E*)-*N*^1^,*N*^2^-*bis*(furan-2-ylmethylene)oxalamide) [45] and [Ni(tenoxicam)(Phen)(H_2_O)_2_](CH_3_COO)_2_·3H_2_O [46]), our complex showed comparable or improved potency against HCT-116, suggesting that different ligands may influence selectivity and, consequently, the modulation of bioactivity.

Moreover, our investigations on the MCF-7 cell line revealed that the complex exhibits efficacy comparable to that of previously reported complexes [Ni(η^2^-NO_3_)(bta)(Phen)], [Ni(η^2^-NO_3_)(btc)(phen)], and [Ni(η^2^-NO_3_)(btf)(Phen)], where bta = 4,4,4-trifluoro-1-phenyl-1,3-butanedionate anion, btc = 1-(4-chlorophenyl)-4,4,4-trifluoro-1,3-butanedionate anion, and btf = 4,4,4-trifluoro-1-(2-furyl)-1,3-butanedionate anion [47].

In agreement with our findings, Mandal et al. [48] previously reported the synthesis of two nickel(II) complexes, [Ni(MPAFA)_2_(H_2_O)_2_]·2Cl·2H_2_O and [Ni(MPAFA)_3_]·2ClO_4_, where MPAFA is the N-(furan-2-ylmethyl)-1-(5-methyl-1H-pyrazol-3-yl) ligand. In these compounds, the Ni(II) ion, in a distorted octahedral coordination environment, induces significant cytotoxicity when coordinated to nitrogen-based heterocyclic ligands. This comparative analysis suggests that the cytotoxic activity can be influenced by coordination geometry, the ligand nature, and the solubility of the complex.

Assays with non-tumor GM07492A cells (Figure 10c) revealed lower complex cytotoxicity (39.8% at 100 μM), demonstrating preferential selectivity for neoplastic cells. This clinically relevant profile suggests a potentially more favorable therapeutic index compared to CP, which showed higher toxicity in healthy cells [49]. However, the moderate cytotoxicity (47.6–65.3% at 100 μM) against tumor MCF-7 and HCT-116 cells indicates that further structural optimization is needed before preclinical development.

As previously discussed in Section 2.1 and Section 2.3, the complex structure revealed several intermolecular interactions, including hydrogen bonds (H···H and O···H/H···O) and π∙∙∙π contacts (centroid distances: ~3.8 Å; dihedral angles < 10°), which enhance its stability and cellular activity. These interactions can facilitate the [Ni(Phen)(Ile)_2_]∙6H_2_O penetration into tumor cells and its binding to intracellular targets, contributing to the observed cytotoxicity in MCF-7 and HCT-116 lines. In general, Phen complexes tend to exhibit a preference for binding sites in cancer cells due to the cellular mutation process, which modifies cell surfaces and enhances interactions with coordination compounds through weak bonds. Moreover, the presence of another organic molecule, such as Ile, amplifies this effect. This mechanism suggests that the complex induces cell death through apoptosis via DNA intercalation, leading to oxidative and structural damage to the double strands. The observed cytotoxicity aligns with reported Phen-based complexes that intercalate DNA, including [Nd(Phen)_2_(NO_3_)_3_] [40], [Cu(Phen)(L-serine)(H_2_O)]NO_3_ [11], [Cu(Phen)(Glycine)Cl]·3H_2_O [10], and [Mg(H_2_O)_3_(Phen)(SO_4_)] [7], oxovanadium(IV) complexes of amino acid Schiff base and Phen [50], among others.

Our results suggest that the main mechanisms of action for [Ni(Phen)(Ile)_2_]·6H_2_O likely differ from those of cisplatin. Preliminary evidence indicates this complex may operate through the generation of reactive oxygen species, interaction with nucleophilic residues of essential proteins, and disruption of cellular redox homeostasis, consistent with observations for other reported complexes [44,51,52,53]. These findings underscore the promising antitumor properties of [Ni(Phen)(Ile)_2_]·6H_2_O, particularly its selective cytotoxicity against cancer cells and favorable therapeutic index compared to cisplatin. While its observed activity highlights its potential as a scaffold for novel anticancer agents, further mechanistic studies (e.g., DNA/protein interaction assays and ROS quantification) are essential to fully elucidate its mode of action and optimize its efficacy.

To complement the biological analyses, an in silico pharmacokinetic study focusing on absorption, distribution, metabolism, and excretion (ADME) properties was conducted. The computational data (Table 3) revealed significant pharmacokinetic differences when comparing the ternary complex [Ni(Phen)(Ile)_2_]·6H_2_O with its analogous compounds, [Ni(Phen)_3_] and [Ni(Ile)_3_], highlighting the former as a promising candidate for pharmaceutical applications. The [Ni(Phen)(Ile)_2_]·6H_2_O complex exhibits a molecular weight of 607.34 g/mol. While this is slightly higher than its analogs, it remains within the acceptable parameters for drug candidates, especially considering that Lipinski’s rule of five allows one violation. Notably, [Ni(Phen)_3_] has a comparable molecular mass (599.31 g/mol) but exhibits a significantly lower total polar surface area (TPSA) of 29.58 Å^2^, indicating poor aqueous solubility. In contrast, our ternary complex, with a TPSA of 117.44 Å^2^, demonstrates an optimal balance between aqueous solubility and membrane permeability, a crucial characteristic for favorable pharmacological performance.

Regarding the lipophilicity and solubility profiles, the [Ni(Phen)(Ile)_2_]·6H_2_O complex shows clear advantages. With an octanol/water partition coefficient (Log *P*_o/w_) of −2.29, the complex is confirmed as hydrophilic and has excellent aqueous solubility (4.60 mg/mL). This characteristic stands in marked contrast to [Ni(Phen)_3_], which is highly lipophilic (Log *P*_o/w_ = 1.56) and practically insoluble (6.11 × 10^−11^ mg/mL) and surpasses [Ni(Ile)_3_], which shows moderate solubility (8.81 × 10^−5^ mg/mL). The superior solubility of the ternary complex is particularly advantageous for developing oral formulations with good bioavailability, representing a significant improvement over the analogous compounds. The high GI absorption (85–90%) and balanced TPSA stem from Ile’s polar carboxylates and the Phen lipophilic ring, enabling both aqueous solubility and membrane permeability, a critical advantage over [Ni(Phen)_3_].

The pharmacokinetic profile further reinforces the advantages of [Ni(Phen)(Ile)_2_]·6H_2_O, as demonstrated by in silico simulations. It exhibits high gastrointestinal absorption (85–90%), significantly outperforming the poorly absorbed [Ni(Phen)_3_]. All compounds share certain characteristics, including no blood–brain barrier (BBB) permeability (reducing the risk of neurotoxicity), being P-glycoprotein substrates, and showing no CYP450 inhibitory activity. Importantly, the [Ni(Phen)(Ile)_2_]·6H_2_O complex displays no toxicity alerts in PAINS/Brenk filters. This favorable profile contrasts with [Ni(Phen)_3_], which shows an alert for polycyclic aromatic hydrocarbons, thereby making [Ni(Phen)(Ile)_2_]·6H_2_O a safer prospective therapeutic candidate. Moreover, its predicted oral bioavailability and the absence of PAINS/Brenk alerts reduce the toxicity risks, positioning [Ni(Phen)(Ile)_2_]·6H_2_O as a lead candidate for further optimization.

The [Ni(Phen)(Ile)_2_]·6H_2_O complex demonstrates a strong profile in terms of drug-likeness assessment and synthetic feasibility. It fully complies with the Veber and Muegge rules and notably outperforms [Ni(Phen)_3_] in the Egan rules, meeting the criteria of major drug-likeness assessments. Furthermore, its synthetic accessibility score (6.73) is more favorable than its analogs, suggesting better practical viability for production. These characteristics, combined with the biological assays previously discussed, position this ternary complex as a promising pharmaceutical candidate. It offers superior aqueous solubility, high gastrointestinal absorption, a clean safety profile, optimal hydrophilic/lipophilic balance, and good synthetic feasibility. These are all critical parameters where [Ni(Phen)(Ile)_2_]·6H_2_O significantly outperforms its analogous compounds for potential drug development.

The antitumor activity of the [Ni(Phen)(Ile)_2_]·6H_2_O complex can be directly related to its structural and electronic properties. The distorted octahedral geometry observed by single-crystal XRD, stabilized through strong hydrogen bonding and π−π stacking interactions, enhances molecular rigidity and facilitates cellular interaction. These supramolecular features, particularly involving the Phen aromatic rings and the Ile carboxyl groups, favor the DNA intercalation process and interaction with redox-sensitive proteins. In addition, DFT calculations show a HOMO–LUMO gap of 4.26 eV (in water) and an electrophilicity index of 4.54 eV. These values indicate a stable electronic configuration, allowing the complex to engage in charge-transfer systems essential for oxidative stress modulation and protein inspection. Moreover, ADME estimations highlight the hydrophilic nature (Log Po/w −2.29) and good solubility (4.60 mg/mL) of the complex. This favorable pharmacokinetic profile, largely due to the dual presence of both nitrogen- and oxygen-donor ligands, suggests efficient gastrointestinal absorption and strong biocompatibility. Ultimately, the inherent structural features of the [Ni(Phen)(Ile)_2_]·6H_2_O complex, including the rigid Phen aromatic core, the hydrogen-bond-rich environment created by the Ile ligands, and the spatial arrangement that enables π−π stacking, act synergistically to confer selective cytotoxicity and pharmacokinetic advantages. The observed correlations between structure and activity reinforce the importance of a precise ligand framework to achieve selective antitumor efficacy.

## 3. Experimental and Theoretical Procedures

### 3.1. Synthesis Process

The [Ni(Phen)(Ile)_2_]∙6H_2_O complex was synthesized using the slow solvent evaporation method from a saturated solution. Initially, a green precipitate was formed by the mixing of an aqueous solution (5 mL) containing 2.5 mmol of nickel nitrate hexahydrate (Sigma Aldrich, >98.5%, St. Louis, MO, USA) with a solution (1 mmol/3 mL) of calcium carbonate (Sigma Aldrich, >99.9%) under magnetic stirring at 320 RPM for 30 min. The resulting product was filtered, washed with deionized water, and dried in an oven for 480 min at 35 °C. Subsequently, a new mixed solution was prepared using 40 mL of deionized water/ethanol (1:1) solvents, where the precipitate was slowly added until fully dissolved along with 2.5 mmol of 1,10-phenanthroline monohydrate (Synth, >99%, Osaka, Japan) and 2.5 mmol of L-isoleucine (Sigma Aldrich, >98.5%). The compounds were homogenized under constant magnetic stirring (360 RPM) at room temperature, resulting in a final purple solution with a pH close to 10. The final solution was then filtered, sealed with plastic wrap with small holes, and placed in an oven at 35 °C for solid-phase nucleation.

### 3.2. Single-Crystal XRD and Structural Solving

A suitable single crystal of the [Ni(Phen)(Ile)_2_]∙6H_2_O complex was selected and analyzed using a Bruker diffractometer (D8 Venture) (Bruker Corporation, Billerica, MA, USA) equipped with an Incoatec CuK_α_ (*λ* = 1.54178 Å) microfocus source. Structural data were collected at 27 °C (300 K) through the Olex2 interface program to the SHELX suite [54]. The structure was determined using the intrinsic phasing method implemented in ShelXT and refined with the ShelXL refinement package via successive full-matrix least-squares cycles [55,56]. The Mercury 2021.3.0 software was employed to generate the crystallographic information file (CIF) and create artwork figures for publication [57]. The CIF file was deposited in the CSD under the code 2453842. Copies of the data can be obtained free of charge via https://www.ccdc.cam.ac.uk/structures/, accessed on 25 June 2025. (Elemental analysis calculated: C—47.45%, H—7.32%, N—9.23%, Ni—9.66%, and O—26.34%.)

### 3.3. Spectroscopic Characterizations

The FT-IR spectrum was collected using a Bruker spectrophotometer (Vertex 70v, Karlsruhe, Germany) with the pastille (2 mg) method, which involved pressing a mixture of 99% potassium bromide (KBr) and 1% powdered [Ni(Phen)(Ile)_2_]∙6H_2_O crystal under 8 tons of pressure for 30 s. The measurement was performed in the spectral range of 400–4000 cm^−1^, with 32 scans and a spectral resolution of 4 cm^−1^.

The Raman spectroscopy measurements were conducted using a Princeton Trivista 557 triple spectrometer equipped with a Charge-Coupled Device (CCD) detector (Horiba, Kyoto, Japan) in a subtractive configuration and thermoelectrically cooled. The excitation source was a helium-neon ion red-laser laser operating at 632.8 nm and a power of 165 mW, with a spectral resolution of 2 cm^−1^.

UV–Vis–NIR absorption spectra were recorded using a double-beam Thermo Scientific Evolution 220 spectrophotometer (Thermo Fisher Scientific, Waltham, MA, USA), covering a broad wavelength range from 190 to 1100 nm. The measurements were performed with a deuterium lamp as the light source and quartz cuvettes with a 0.1 cm path length.

### 3.4. DFT Calculations

Computational calculations were performed employing the DFT method for geometry optimization and the analysis of geometric, electronic, and vibrational properties using the Gaussian 16 software [58]. The initial coordinates of the complex were extracted from the single-crystal XRD experiment (2453842). The geometry of the [Ni(Phen)(Ile)_2_]∙complex was optimized using the PBE1PBE functional [59], associated with the 6-311++G(d,p) basis set for H, N, C, and O atoms, and the Stuttgart−Dresden (SDD) pseudopotential was used for representing core electrons of the Ni atom, while basis functions of the SDD basis set were also used for valence electrons of the metal center [60]. The hybrid PBE1PBE functional was selected due to its accuracy for the study of complexes involving first-row transition metals, as previously reported in studies of coordination compounds [61]. Additionally, the solvation effect was considered using the integral equation formalism of the polarizable continuum model (IEFPCM) in methanol (*ε* = 32.61) and water (*ε* = 78.35) [62]. The electrostatic potential surface (EPS) maps were also calculated from the optimized geometries of the complex. In addition, the vibrational frequencies were calculated, confirming each optimized geometry as a minimum on the potential energy surface, as all calculated frequencies are positive. The analysis of vibrational modes was conducted using the free program vibAnalysis and the Chemcraft 1.8 software, employing the Bayesian regression method to determine the relevance of the vibrational modes [63,64]. Additionally, the calculated IR spectra were adjusted by a scaling factor of 0.9594, while the Raman spectra were corrected using three distinct factors, 0.9594 (200–700 cm^−1^), 0.9748 (700–1800 cm^−1^), and 0.9812 (2800–3200 cm^−1^), to achieve a better correlation between the experimental and theoretical data [38].

The root mean square deviation (RMSD) was used to analyze the differences between the experimental geometric data and the theoretical results obtained from the computational calculations. This metric quantifies discrepancies in the atomic positions of a given complex and is widely applied in computational chemistry to evaluate the accuracy of the theoretical models [65]. Table A4 compares bond lengths and angles in three distinct environments (vacuum, water, and methanol) based on atomic coordinates, molecular superposition, and the difference between optimized geometries. The RMSD was calculated using the following equation: RMSD = $\sqrt{\frac{1}{N}\sum_{i=1}^{N}{\left(di\right)}^{2}}$, where N = total number of atoms, and *d*_i_ = difference in bond lengths/angles between the compared structures.

### 3.5. Hirshfeld Surfaces, Crystal Void Studies, and ADME

Hirshfeld surfaces [66], 2D fingerprint plots [67,68], and crystal voids were calculated using the CrystalExplorer 17 software [69] to analyze intermolecular interactions in the system and identify the percentage of free space in the unit cell. The Hirshfeld surfaces were mapped based on the normalized distance (*d*_norm_) in terms of distances from a point on the surface to the external (*d*_e_) and internal (*d*_i_) atoms, as well as the van der Waals radius (*r*_vdW_). The 2D fingerprint plots were generated from the de and *d*_i_ functions, encompassing all intermolecular contacts in the structural lattice to quantify each interaction. Additionally, the voids in the primitive unit cell were calculated from isosurfaces of procrystal electron density (0.002 a.u.). Complementarily, the in silico pharmacokinetic properties via ADME were evaluated using the SwissADME tool (free version) based on the optimized molecular geometry of [Ni(Phen)(Ile)_2_]·6H_2_O and related compounds for comparative analyses.

### 3.6. In Vitro Cytotoxicity Assay

A cell viability assay was conducted to evaluate the cytotoxicity of the [Ni(Phen)(Ile)_2_]∙6H_2_O complex using two distinct tumor cell lines: MCF-7 (human mammary adenocarcinoma) and HCT-116 (human colorectal cancer). The data collected from these cancer cells were compared with a non-tumor cell line (GM07492A—lung fibroblasts) to assess the selectivity effect of the compound. The cells were cultured following the method described by Supino in Dulbecco’s Modified Eagle Medium (DMEM) [70,71]. Each cell line was seeded in 96-well plates (1 × 10^4^ cells in 100 μL/well) for 24 and 48 h and treated with 10, 50, and 100 μM (100 μL per well) concentrations of the powdered crystal. The same process was applied to the cisplatin (Sigma Aldrich, >99.9%) complex (100 μM) for comparison. The cytotoxicity of the samples was evaluated using the 3-(4,5-dimethylthiazol-2-yl)-2,5-diphenyltetrazolium bromide (MTT) test by adding 10 μL to each well after the contact times between the cells and the complex. Subsequently, the cells were incubated for 180 min in a light-free environment, and the resulting crystals were solubilized in dimethylsulfoxide. The samples were then read using a Spectra Max 190 spectrophotometer (Molecular Devices, San Jose, CA, USA) at 540 nm [69,71].

## 4. Conclusions

The novel ternary nickel(II) complex [Ni(Phen)(Ile)_2_]∙6H_2_O was successfully synthesized and characterized, demonstrating significant potential as an antitumor agent. A structural analysis via single-crystal XRD revealed a monoclinic system with octahedral Ni(II) coordination, stabilized by intermolecular hydrogen bonds and *π*−*π* interactions, as confirmed by a Hirshfeld surface analysis. DFT calculations provided insights into the complex thermodynamic stability, electronic properties, and solvation effects, with Gibbs free energy values indicating spontaneous formation and favorable solute–solvent interactions in aqueous and methanolic environments. Spectroscopic studies (FT-IR and Raman) aligned well with theoretical predictions, validating the fundamental vibrational modes and functional group interactions. The HOMO−LUMO gap and electrophilicity index highlighted its charge-transfer potential, suitable for biological applications. UV–Vis–NIR spectroscopic data demonstrated that the complex is stable in solution and does not undergo ionic dissociation. In vitro cytotoxicity assays against MCF-7 and HCT-116 cancer cell lines revealed dose-dependent activity, with notable selectivity and reduced toxicity toward non-tumor GM07492A cells compared to cisplatin. The [Ni(Phen)(Ile)_2_]·6H_2_O complex exhibits compelling characteristics that are indicative of promising antitumor potential. This is supported by its distinctive structural features, selective cytotoxicity, and favorable in silico pharmacokinetic profile. Moreover, it exhibits an optimal balance of solubility, absorption, and drug-likeness, as well as a clean safety profile. While these encouraging findings are preliminary and require further studies before preclinical exploration, they strongly suggest their potential as a scaffold for developing novel anticancer agents. This work contributes to the growing field of metal-based therapeutics, offering a foundation for developing advanced anticancer agents with improved selectivity and safety.

## Figures and Tables

**Table 1:** Crystal data and structure refinement for the [Ni(Phen)(Ile)_2_]∙6H_2_O complex.

Empirical formula	C_24_H_44_N_4_NiO_10_
Formula weight [g.mol^−1^ ]	607.34
Temperature [K]	300
Crystal system	monoclinic
Space group	*C*2
*a* [Å]	18.0810(1)
*b* [Å]	10.1111(1)
*c* [Å]	10.0475(1)
α [°]	90
*β* [°]	122.137(2)
*γ* [°]	90
Volume [Å^3^]	1555.4(3)
*Z*	2
*ρ*_calc_ [g/cm^3^]	1.297
*μ* [mm^−1^]	1.373
F (000)	648.0
Crystal size [mm^3^]	0.697 × 0.512 × 0.318
Radiation	CuKα (*λ* = 1.54178)
2Θ range for data collection [°]	17.66 to 144.42
Index ranges	−22 ≤ h ≤ 22, −12 ≤ k ≤ 12, −12 ≤ l ≤ 12
Reflections collected	15,759
Independent reflections	2940 [R_int_ = 0.0340, R_sigma_ = 0.0300]
Data/restraints/parameters	2940/93/253
Goodness-of-fit on F^2^	1.043
Final R indexes [I ≥ 2σ (I)]	R_1_ = 0.0274, wR_2_ = 0.0709
Final R indexes [all data]	R_1_ = 0.0277, wR_2_ = 0.0713
Largest diff. peak/hole/e Å^−3^	0.17/−0.15
Flack parameter	0.16 (3)

CSD 2453842 contains supplementary crystallographic data. The data can be obtained free of charge from https://www.ccdc.cam.ac.uk/structures/, accessed on 25 June 2025.

**Table 2:** Chemical reactivity parameters (in eV) for the [Ni(Phen)(Ile)_2_] complex calculated at the PBE1PBE/6-311++G(d,p)/SDD level in different media.

Descriptors	*E*_HOMO_ [eV]	*E*_LUMO_ [eV]	*Gap* [eV]	*IP* [eV]	*EA* [eV]	*η* [eV]	*ς* [eV^−1^]	*μ* [eV]	*χ* [eV]	*ϖ* [eV]
Vacuum	−5.94	−2.74	3.20	5.94	2.74	1.60	0.80	−4.34	4.34	5.88
Water	−6.53	−2.27	4.26	6.53	2.27	2.13	1.06	−4.40	4.40	4.54
Methanol	−6.51	−2.28	4.22	6.51	2.28	2.11	1.06	−4.40	4.40	4.58

**Table 3:** ADME pharmacokinetic parameters and drug-likeness properties for the [Ni(Phen)(Ile)_2_]·6H_2_O complex and related compounds.

	[Ni(Phen)(Ile)_2_]∙6H_2_O	[Ni(Phen)_3_]	[Ni(Ile)_3_]
Physicochemical properties			
Molecular weight (g/mol)	607.34	599.31	460.19
TPSA (Å^2^)	117.44	29.58	114.99
Lipophilicity			
Log P_o/w_ (SILICOS-IT)	–2.29	1.56	0.78
Water Solubility			
Log S (SILICOS-IT)	–2.05	–10.21	–4.05
Solubility (mg/mL)	4.60	6.11 × 10^−11^	8.81 × 10^−5^
Class	Soluble	Insoluble	Moderately soluble
Pharmacokinetics			
GI absorption	High	Low	High
BBB permeant	No	No	No
P-gp substrate	Yes	Yes	Yes
CYP1A2 inhibitor	No	No	No
CYP2C19 inhibitor	No	No	No
CYP2C9 inhibitor	No	No	No
CYP2D6 inhibitor	No	No	No
CYP3A4 inhibitor	No	No	No
LogKp (skin permeation) (cm/s)	–6.55	–5.15	–5.72
Drug-likeness			
Lipinski	Yes; 1 violation *	Yes; 1 violation *	Yes; 0 violation
Ghose	No	No	Yes
Veber	Yes	Yes	Yes
Egan	Yes	No	Yes
Muegge	Yes	No	Yes
Bioavailability Score	0.55	0.55	0.55
Medicinal chemistry			
PAINS	0 alert	0 alert	0 alert
Brenk	0 alert	1 alert **	0 alert
Lead-likeness	No	No	No; 2 violations ^#^
Synthetic accessibility	6.73	4.04	5.91

* MW > 500; ** polycyclic_aromatic_hydrocarbon_3; ^#^ MW > 350 and XLOGP3 > 3.5.

## Data Availability

The crystal structure information is available at www.ccdc.cam.ac.uk. Cambridge Structural Database under the numbers 240117.

## Supplementary Materials

The following supporting information can be downloaded at: https://www.mdpi.com/article/10.3390/molecules30132873/s1, Figure S1: Refined XRD pattern of powdered [Ni(Phen)(Ile)_2_]∙6H_2_O crystal at room conditions.

## Appendix A

**Table A1:** Bond lengths of the symmetrical unit for the crystal [Ni(Phen)(Ile)_2_]∙6H_2_O.

Atom	Atom	Length (Å)	Atom	Atom	Length (Å)
Ni01	O1′	2.054(2)	C10	C9	1.395(1)
Ni01	O1	2.054(2)	C6	C7	1.394(1)
Ni01	N3	2.082(1)	C6	C5	1.430(1)
Ni01	N3′	2.082(1)	C8	C9	1.364(1)
Ni01	N2	2.132(8)	C8	C7	1.402(1)
Ni01	N1	2.080(1)	C7	C11	1.399(1)
O1	C13	1.258(3)	C11	C12	1.460(4)
N3	C14	1.479(3)	N1	C1	1.330(1)
O2	C13	1.236(3)	N1	C5	1.353(1)
C13	C14	1.534(3)	C1	C2	1.378(1)
C14	C15	1.532(3)	C2	C3	1.360(2)
C17	C18	1.521(4)	C3	C4	1.405(1)
C17	C15	1.518(5)	C4	C5	1.402(9)
N2	C10	1.336(9)	C4	C12	1.380(2)
N2	C6	1.345(1)	C15	C16	1.529(4)

**Table A2:** Bond angles of the symmetrical unit for the crystal [Ni(Phen)(Ile)_2_]∙6H_2_O.

Atom	Atom	Atom	Angle (˚)	Atom	Atom	Atom	Angle (˚)
O1′	Ni01	O1	95.74(14)	N2	C10	C9	122.20(8)
O1	Ni01	N3	80.36(8)	N2	C6	C7	123.30(8)
O1	Ni01	N3′	93.21(8)	N2	C6	C5	116.80(7)
O1′	Ni01	N3	93.21(8)	C7	C6	C5	119.90(8)
O1′	Ni01	N3′	80.36(8)	C9	C8	C7	121.30(8)
O1′	Ni01	N2	176.20(3)	C8	C9	C10	118.20(8)
O1	Ni01	N2	86.20(2)	C6	C7	C8	116.20(9)
O1′	Ni01	N1	100.10(3)	C6	C7	C11	120.10(11)
O1	Ni01	N1	163.60(3)	C11	C7	C8	123.70(12)
N3	Ni01	N3′	170.47(14)	C7	C11	C12	119.80(10)
N3	Ni01	N2	90.40(3)	C1	N1	Ni01	126.80(8)
N3′	Ni01	N2	96.20(3)	C1	N1	C5	118.90(9)
N1	Ni01	N3	94.40(3)	C5	N1	Ni01	114.20(6)
N1	Ni01	N3′	93.70(3)	N1	C1	C2	122.70(12)
N1	Ni01	N2	78.30(4)	C3	C2	C1	118.90(13)
C13	O1	Ni01	116.85(16)	C2	C3	C4	120.60(9)
C14	N3	Ni01	110.56(14)	C5	C4	C3	116.70(9)
O1	C13	C14	117.80(2)	C12	C4	C3	122.80(1)
O2	C13	O1	123.50(2)	C12	C4	C5	120.50(15)
O2	C13	C14	118.50(2)	N1	C5	C6	117.40(6)
N3	C14	C13	109.47(18)	N1	C5	C4	122.30(8)
N3	C14	C15	113.76(19)	C4	C5	C6	120.30(7)
C15	C14	C13	113.98(19)	C4	C12	C11	119.40(1)
C15	C17	C18	114.30(4)	C17	C15	C14	112.50(2)
C10	N2	Ni01	128.00(6)	C17	C15	C16	112.60(3)
C10	N2	C6	118.80(7)	C16	C15	C14	110.00(2)
C6	N2	Ni01	113.10(5)				

**Table A3:** Hydrogen bonding observed in the crystal structure of the complex [Ni(Phen)(Ile)_2_]∙6H_2_O.

Contacts	Distance [Å]	Angle [°]
O1∙∙∙H–O *	2.035	160.14
O2∙∙∙H–O *	1.954	171.50
H–O∙∙∙H^+^	1.838	121.92
H∙∙∙O–H^+^	2.024	129.39

* Atoms O1 and O2 originate from the carboxylate group of the Ile ligand, while the H–O moiety derives from coordinated H_2_O molecules. ^+^ Interactions involving free H_2_O molecules.

**Table A4:** Theoretical and experimental data for the bond lengths and angles of the [Ni(Phen)(Ile)_2_] complex calculated with the PBE1PBE functional in vacuum, water, and methanol.

**Bond lengths [Å]**							
**Atoms**	**Vacuum**		**Water**		**Methanol**		**Exp.**
	**Calc.**	**RMSD**	**Calc.**	**RMSD**	**Calc.**	**RMSD**	
Ni01–O1′	2.01	0.0296	2.05	0.0021	2.05	0.0035	2.05
Ni01–O1	2.01	0.0296	2.05	0.0021	2.05	0.0035	2.05
Ni01–N3	2.10	0.0134	2.10	0.0098	2.10	0.0106	2.08
Ni01–N3′	2.10	0.0141	2.10	0.0106	2.10	0.0106	2.08
Ni01–N2	2.15	0.0113	2.13	0	2.12	0.0070	2.13
Ni01–N1	2.15	0.0480	2.12	0.0296	2.12	0.0296	2.08
O1–C13	1.28	0.0183	1.27	0.0106	1.27	0.0106	1.26
N3–C14	1.48	0.0014	1.47	0.0035	1.47	0.0035	1.48
O2–C13	1.22	0.0098	1.24	0	1.24	0	1.24
C13–C14	1.55	0.0113	1.55	0.0077	1.55	0.0077	1.53
C14–C15	1.53	0.0014	1.54	0.0021	1.53	0.0014	1.53
C17–C18	1.52	0.0014	1.52	0.0021	1.52	0.0021	1.52
C17–C15	1.53	0.0098	1.53	0.0098	1.53	0.0098	1.52
N2–C10	1.32	0.0113	1.32	0.0106	1.32	0.0106	1.34
N2–C6	1.35	0.0014	1.35	0.0028	1.35	0.0028	1.35
C10–C9	1.40	0.0070	1.40	0.0049	1.40	0.0049	1.40
C6–C7	1.41	0.0091	1.41	0.0091	1.41	0.0091	1.39
C6–C5	1.44	0.0049	1.44	0.0035	1.44	0.0035	1.43
C8–C9	1.38	0.0077	1.38	0.0077	1.38	0.0077	1.36
C8–C7	1.41	0.0042	1.41	0.0035	1.41	0.0035	1.40
C7–C11	1.43	0.0226	1.43	0.0226	1.43	0.0226	1.40
C11–C12	1.36	0.0721	1.36	0.0721	1.36	0.0721	1.46
N1–C1	1.32	0.0070	1.32	0.0063	1.32	0.0063	1.33
N1–C5	1.35	0.0042	1.35	0.0028	1.35	0.0028	1.35
C1–C2	1.40	0.0183	1.40	0.0183	1.40	0.0169	1.38
C2–C3	1.38	0.0106	1.38	0.0106	1.38	0.0106	1.36
C3–C4	1.41	0.0021	1.41	0.0014	1.41	0.0014	1.41
C4–C5	1.41	0.0035	1.41	0.0035	1.41	0.0035	1.40
C4–C12	1.43	0.0360	1.43	0.0360	1.43	0.0360	1.38
C15–C16	1.53	0.0021	1.53	0.0021	1.53	0.0021	1.53
**Bond angles [°]**							
**Atoms**	**Vacuum**		**Water**		**Methanol**		**Exp.**
	**Calc.**	**RMSD**	**Calc.**	**RMSD**	**Calc.**	**RMSD**	
O1′–Ni01–O1	106.20	7.3963	96.14	0.2828	96.54	0.5657	95.74
O1–Ni01–N3	81.22	0.6081	79.69	0.4738	79.69	0.4738	80.36
O1–Ni01–N3′	86.92	4.4477	91.18	1.4354	90.91	1.6263	93.21
O1′–Ni01–N3	86.88	4.4760	91.18	1.4354	90.92	1.6193	93.21
O1′–Ni01–N3′	81.21	0.6010	79.68	0.4808	79.68	0.4808	80.36
O1′–Ni01–N2	165.00	7.9196	169.90	4.4548	169.77	4.5467	176.20
O1–Ni01–N2	88.60	1.6971	92.02	4.1154	92.84	4.6952	86.20
O1′–Ni01–N1	88.58	8.1459	93.02	5.0063	93.83	4.4336	100.10
O1–Ni01–N1	165.03	1.0112	169.90	4.4548	169.77	4.3628	163.60
N3–Ni01–N3’	160.18	7.2761	166.39	2.8850	165.93	3.2103	170.47
N3–Ni01–N2	97.60	5.0912	95.94	3.9174	96.14	4.0588	90.40
N3′–Ni01–N2	97.88	1.1879	94.60	1.1314	94.76	1.0182	96.20
N1–Ni01–N3	97.90	2.4749	94.61	0.1485	94.77	0.2616	94.40
N1–Ni01–N3′	97.61	2.7648	95.94	1.5839	95.94	1.5839	93.70
N1–Ni01–N2	76.73	1.1102	78.17	0.0919	78.12	0.1273	78.30
C13–O1–Ni01	117.01	0.1131	116.37	0.3394	116.34	0.3606	116.85
C14–N3–Ni01	105.13	3.8396	107.95	1.8455	107.64	2.0648	110.56
O1–C13–C14	114.31	2.4678	116.37	1.0112	116.25	1.0960	117.80
O2–C13–O1	126.22	1.9233	125.13	1.1526	125.17	1.1809	123.50
O2–C13–C14	119.42	0.6505	118.43	0.0495	118.52	0.0141	118.50
N3–C14–C13	109.67	0.1414	110.13	0.4667	110.13	0.4667	109.47
N3–C14–C15	114.59	0.5869	114.58	0.5798	114.62	0.6081	113.76
C15–C14–C13	113.51	0.3323	113.24	0.5233	113.31	0.4738	113.98
C15–C17–C18	113.85	0.3182	113.86	0.3111	113.87	0.3041	114.30
C10–N2–Ni01	126.39	1.1384	127.60	0.2828	127.57	0.3041	128.00
C10–N2–C6	119.11	0.2192	118.82	0.0141	118.83	0.0212	118.80
C6–N2–Ni01	114.48	0.9758	113.56	0.3253	113.59	0.3465	113.10
C7–C6–C5	119.80	0.0707	119.72	0.1273	119.73	0.1202	119.90
C9–C8–C7	119.53	1.2516	119.46	1.3011	119.46	1.3011	121.30
C8–C9–C10	119.32	0.7920	119.16	0.6788	119.17	0.6859	118.20
C6–C7–C8	116.85	0.4596	117.08	0.6223	117.07	0.6152	116.20
C6–C7–C11	119.11	0.7000	119.28	0.5798	119.27	0.5869	120.10
C11–C7–C8	124.03	0.2333	123.63	0.0495	123.64	0.0424	123.70
C7–C11–C12	121.08	0.9051	120.98	0.8344	120.99	0.8415	119.80
C1–N1–Ni01	126.39	0.2899	127.60	0.5657	127.56	0.5374	126.80
C1–N1–C5	119.10	0.1414	118.82	0.0566	118.83	0.0495	118.90
C5–N1–Ni01	114.49	0.2051	113.56	0.4525	113.59	0.4313	114.20
N1–C1–C2	122.11	0.4172	122.50	0.1414	122.52	0.1273	122.70
C3–C2–C1	119.32	0.2970	119.16	0.1838	119.17	0.1909	118.90
C2–C3–C4	119.53	0.7566	119.46	0.8061	119.46	0.8061	120.60
C5–C4–C3	116.85	0.1061	117.08	0.2687	117.07	0.2616	116.70
C12–C4–C3	124.03	0.8697	123.63	0.5869	123.64	0.5940	122.80
C12–C4–C5	119.11	0.9829	119.28	0.8627	119.27	0.8697	120.50
N1–C5–C6	117.14	0.1838	117.35	0.0354	117.34	0.0424	117.40
N1–C5–C4	123.06	0.5374	122.92	0.4384	122.92	0.4384	122.30
C4–C5–C6	119.79	0.3606	119.72	0.4101	119.73	0.4031	120.30
C4–C12–C11	121.08	1.1879	120.98	1.1172	120.99	1.1243	119.40
C17–C15–C14	112.15	0.2475	112.08	0.2970	112.09	0.2899	112.50
C17–C15–C16	112.37	0.1626	112.32	0.1980	112.31	0.2051	112.60
C16–C15–C14	111.27	0.8980	111.04	0.7354	111.05	0.7425	110.00

**Table A5:** Theoretical and experimental FT-IR and Raman vibrational modes for the [Ni(Phen)(Ile)_2_]∙6H_2_O.

ω_IR_ [cm^−1^]	ω_Raman_ [cm^−1^]	ω_calcvac_ [cm^−1^]	ω_calcwat_ [cm^−1^]	ω_calmet_ [cm^−1^]	Assignments_vac_	Assignments_wat_	Assignments_met_
3479	-	3459	3458	3457	*ν_a_*(H1N1H2)(77)	*ν_a_*(H1N1H2)(88)	*ν_a_*(H1N1H2)(92)
3361	-	3380	3385	3384	*ν_s_*(H1N1H2)(42) + *ν_s_*(H21N4H22)(48)	*ν_s_*(H1N1H2)(46) + *ν_s_*(H21N4H22)(48)	*ν_s_*(H1N1H2)(45) + *ν_s_*(H21N4H22)(48)
**-**	3110	3098	3101	3101	*ν*(H11C11)(28) + *ν*(H8C7)(25)	*ν*(H11C11)(23) + *ν*(H8C7)(23)	*ν*(H11C11)(15) + *ν*(H8C7)(24)
3058	3067	3058	3075	3075	*ν*(H10C10)(27) + *ν*(H6C4)(25)	*ν*(H10C10)(17) + *ν*(H6C4)(18) + *ν*(H7C6)(15) + *ν*(H12C12)(15)	*ν*(H10C10)(19) + *ν*(H6C4)(18) + *ν*(H7C6)(16) + *ν*(H12C12)(17)
2964	3012	2991	2980	2988	*ν*(H26C22)(16)+ *ν*(H14C16)(13)+ *ν*(H27C22)(11)	*ν_a_*(H14C16H16)(32)+ *ν_a_*(H26C22H28)(25)	*ν_a_*(H26C22H28)(53)
**-**	2943	2947	2949	2949	*ν*(H17C17)(21)+ *ν*(H29C23)(10)	*ν*(H29C23)(19)+ *ν*(H24C21)(16)	*ν*(H17C17)(17)+ *ν*(H4C3)(14)
**-**	2935	2940	2937	2937	*ν*(H23C20)(33)+ *ν*(H3C2)(10)+ *ν*(H29C23)(11)	*ν*(H23C20)(15)+ *ν*(H24C21)(13)+ *ν*(H29C23)(10)	*ν*(H23C20)(15)+ *ν*(H3C2)(12)+ *ν*(H29C23)(12)
2914	2917	2922	2919	2919	*ν_s_*(H27C22H28)(43)+ *ν_s_*(H15C16H16)(39)	*ν_s_*(H27C22H28)(36)+ *ν_s_*(H15C16H16)(32)	*ν_s_*(H27C22H28)(29)+ *ν_s_*(H15C16H16)(39)
2881	2880	2906	2913	2913	*ν_s_*(H18C18H20)(55)	*ν_s_*(H30C24H32)(46)	*ν_s_*(H30C24H32)(44)
2848	2875	2875	2896	2895	*ν_s_*(H24C21H25)(43)	*ν_s_*(H24C21H25)(60)	*ν_s_*(H24C21H25)(64)
1660	1630	1690	1614	1615	*ν*(C19O4)(36)+ *ν*(C1O2)(35)+ *ν*(C19O3)(10)+ *ν*(C1O1)(10)	*ν*(C9C15)(14)	*ν*(C9C15)(14)+ *ν*(C19O4)(9)+ *ν*(C1O2)(9)
1581	1610	1679	1592	1594	*ν*(C1O2)(35)+ *ν*(C19O4)(35)+ *ν*(C19O3)(10)+ *ν*(C1O1)(10)	*ν*(C19O4)(25)+ *ν*(C1O2)(25)	*ν*(C19O4)(24)+ *ν*(C1O2)(24)
1517	1521	1507	1510	1510	*δ*(phen_ring_)(55)	*δ*(phen_ring_)(55)	*δ*(phen_ring_)(54)
1456	1457	1481	1481	1480	*γ*(H11C11C12)(22) + *γ*(C10C11C12)(10)	*γ*(H11C11C12)(20) + *γ*(C10C11C12)(9)	*γ*(H11C11C12)(23) + *γ*(C10C11C12)(9)
1405	1429	1406	1404	1404	*δ*(phen_ring_)(35)	*δ*(phen_ring_)(21)	*δ*(phen_ring_)(22)
1348	1349	1357	1370	1370	*ν*(C50O45)(9)+ *ν*(C19O3)(9)	*ν*(C1O1)(12)+ *ν*(C19O3)(11)	*ν*(C1O1)(12)+ *ν*(C19O3)(11)
1286	1309	1320	1325	1325	*ν*(C1O1)(12)	*γ*(O1C1O2)(11)	*γ*(O1C1O2)(11)
1259	1262	1253	1253	1253	*γ*(H5C3C16)(9) + *γ*(H25C21C22)(9)	*γ*(H25C21C22)(9)+ *γ*(H5C3C16)(9)	*γ*(H25C21C22)(9)+ *γ*(H5C3C16)(9)
1218	1204	1228	1220	1223	*γ*(H17C17C18)(9) + *γ*(H29C23C24)(9)	*γ*(H3C2C17)(9)	*γ*(H23C20C19)(9)
1172	1158	1157	1157	1157	*δ*(C3C17C18)(9)	*δ*(C3C17C18)(9)	*δ*(C3C17C18)(9)
1147	1147	1143	1144	1145	*γ*(N1C2C17)(9)	*γ*(N1C2C17)(9)	*γ*(N1C2C17)(9)
1114	1115	1117	1117	1117	*ν*(C23C24)(9)	*ν*(C23C24)(9)	*ν*(C23C24)(9)
1072	1057	1083	1083	1083	*δ*(C16C3C17)(9)	*δ*(C16C3C17)(9)	*δ*(C16C3C17)(9)
1012	1016	1033	1034	1034	*ν*(C4C7)(11) + *ν*(C10C11)(10)	*ν*(C3C16)(11)	*ν*(C4C7)(11)
987	998	1018	1025	1025	*ν*(C3C16)(9)	*ν*(C3C16)(9)	*ν*(C3C16)(9)
960	963	957	961	961	*ν*(C23C24)(9)	*ν*(C23C24)(9)	*ν*(C23C24)(9)
891	874	921	925	925	*ν*(C1C2)(9)+ *ν*(O1C1)(9)	*ν*(C1C2)(9)+ *ν*(O1C1)(9)	*ν*(C1C2)(9)+ *ν*(O1C1)(9)
848	830	829	829	829	*δ*(Fen_ring_)(63)	*δ*(Fen_ring_)(61)	*δ*(Fen_ring_)(64)
806	813	779	774	774	*δ*(Ile_ring_)(25)	*δ*(Ile_ring_)(24)	*δ*(Ile_ring_)(30)
725	733	715	713	713	*δ*(Fen_ring_)(55)	*δ*(Fen_ring_)(69)	*δ*(Fen_ring_)(73)
667	652	639	644	644	*δ*(Ile_ring_)(28)	*δ*(Ile_ring_)(9)	*δ*(Ile_ring_)(9)
**-**	614	620	622	622	*δ*(Fen_ring_)(69)	*ω*(N3C7C8)(9)	*ω*(N3C7C8)(9)
595	596	574	585	586	*τ*(N4Ni1N1C2)(9) + *τ*(N1Ni1N4C20)(9)	*ρ*(N4C20C19)(9)	*ρ*(N4C20C19)(9)
567	564	550	566	566	*ω*(O4C19C20)(9) + *ω*(O1C1C2)(9)	*τ*(N4C20C19O3)(9)	*ν*(C3C17)(9)
513	514	504	495	499	*δ*(Fen_ring_)(52)	*δ*(Fen_ring_)(56)	*δ*(Fen_ring_)(65)
486	489	484	484	484	*ρ*(Ni1O1C1)(9) *τ*(Ni1O3C19)(9)	*τ*(Ni1N2C5C8)(9)	*ρ*(O1C1C2)(9)
453	451	438	424	428	*τ*(Fen_ring_)(35)	*ν*(Ni1N1)(9)	*ν*(Ni1N1)(9)
420	429	415	410	412	*b_r_*(Fen_ring_)(21)	*b_r_*(Fen_ring_)(21)	*b_r_*(Fen_ring_)(31)
**-**	384	393	385	385	*τ*(O1Ni1O3)(9)	*τ*(O1Ni1O3)(9)	*τ*(O1Ni1O3)(9)
**-**	372	373	368	368	*ν*(Ni1N1)(9)	*ν*(Ni1N1)(9)	*ν*(Ni1N1)(9)
**-**	298	308	302	303	*δ*(Fen_ring_)(38)	*δ*(Fen_ring_)(38)	*δ*(Fen_ring_)(38)
**-**	272	287	280	281	*τ*(C14N3Ni1)(9)	*τ*(C14N3Ni1)(9)	*τ*(C14N3Ni1)(9)
**-**	246	261	263	263	*δ*(Ile_ring_)(19)	*δ*(Ile_ring_)(19)	*δ*(Ile_ring_)(19)
**-**	224	237	238	238	*ν*(Ni1N1)(9)	*ν*(Ni1N1)(9)	*ν*(Ni1N1)(9)
**-**	206	201	207	206	*δ*(Ile_ring_)(9)	*δ*(Ile_ring_)(9)	*δ*(Ile_ring_)(9)
	185					Lattice mode	
	172						
	165						
	138						
	108						
	92						
	85						
	67						

Theoretical spectrum (*ω*calc)/experimental FT-IR (*ω*IR)/experimental Raman (*ω*RAMAN). Nomenclature: *ν* = stretching; *ν_s_* = symmetric stretching; *ν_a_* = antisymmetric stretching; *ρ* = rocking; *δ* = bending; *γ* = scissoring; *ω* = wagging; *tw* = twisting; *τ* = torsion; *b_r_* = breathing.
