# Supplementary material for: Exploring the Potential of a New Nickel(II):Phenanthroline Complex with L-isoleucine as an Antitumor Agent: Design, Crystal Structure, Spectroscopic Characterization, and Theoretical Insights
Source: Molecules. 2025 Jul 6;30(13):2873. doi: 10.3390/molecules30132873 (PMC12251309; doi:10.3390/molecules30132873)
Supplement: Supplementary file 1 [file molecules-30-02873-s001.zip › molecules-3694273-supplementary.pdf]

molecules

MDPI

Supplementary Material

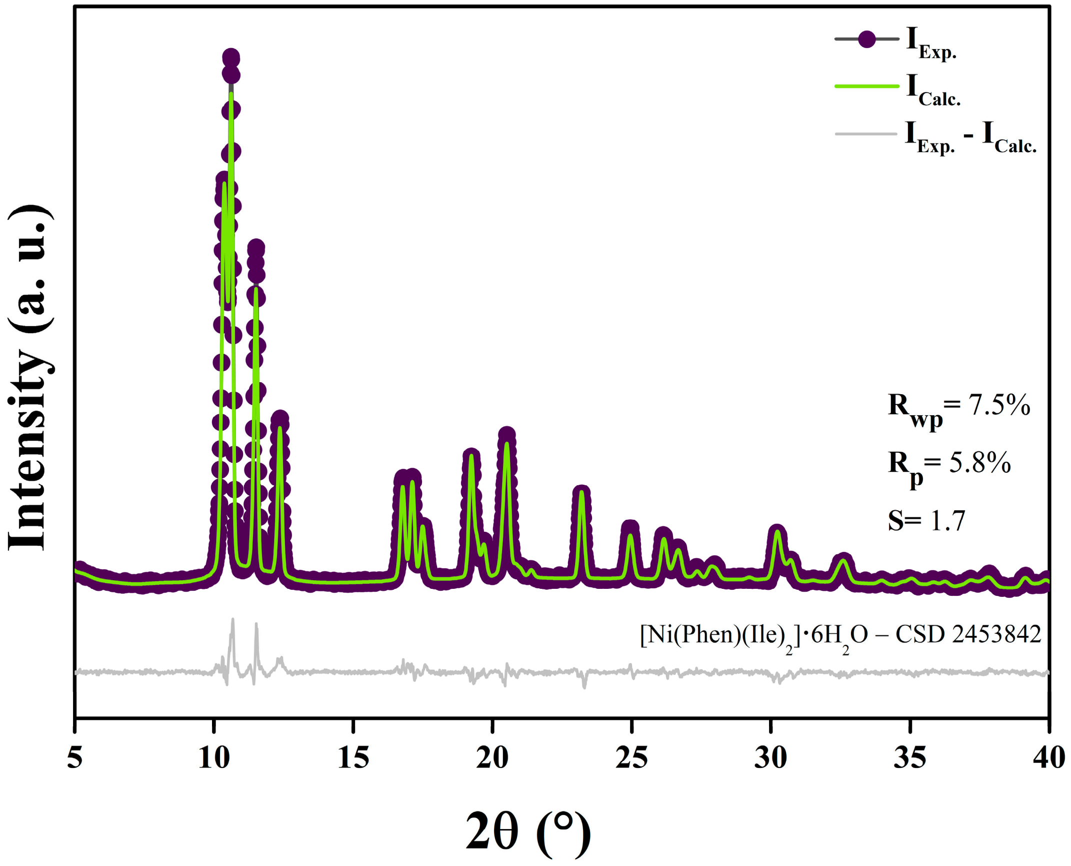

Figure S1. Refined XRD pattern of powdered $[Ni(Phen)(Ile)_2]\cdot 6H_2O$ crystal at room conditions.
